# Using Decision Models to Enhance Investigations of Individual Differences in Cognitive Neuroscience

**DOI:** 10.3389/fpsyg.2016.00081

**Published:** 2016-02-09

**Authors:** Corey N. White, Ryan A. Curl, Jennifer F. Sloane

**Affiliations:** Department of Psychology, Syracuse UniversitySyracuse, NY, USA

**Keywords:** drift–diffusion model, linear ballistic accumulator model, fMRI, EEG, individual differences

## Abstract

There is great interest in relating individual differences in cognitive processing to activation of neural systems. The general process involves relating measures of task performance like reaction times or accuracy to brain activity to identify individual differences in neural processing. One limitation of this approach is that measures like reaction times can be affected by multiple components of processing. For instance, some individuals might have higher accuracy in a memory task because they respond more cautiously, not because they have better memory. Computational models of decision making, like the drift–diffusion model and the linear ballistic accumulator model, provide a potential solution to this problem. They can be fitted to data from individual participants to disentangle the effects of the different processes driving behavior. In this sense the models can provide cleaner measures of the processes of interest, and enhance our understanding of how neural activity varies across individuals or populations. The advantages of this model-based approach to investigating individual differences in neural activity are discussed with recent examples of how this method can improve our understanding of the brain–behavior relationship.

## Introduction

Researchers in cognitive neuroscience have placed recent emphasis on relating differences in brain activity to cognitive performance in service of identifying individual or group differences in neural processing. For example, Tam et al. (2015) related reaction times (RTs) from a Stroop task to blood oxygen level-dependent (BOLD) activity from functional magnetic resonance imaging (fMRI). They showed that longer RTs were associated with greater activity in frontoparietal areas for older adults, but they were associated with greater activity in default mode network areas for younger adults. Studies such as these can provide insight into how the neural mechanisms driving behavior differ across individuals or groups. This approach can be generalized to investigate individual differences from a range of tasks (e.g., memory, perceptual, emotional, etc.) and used to compare neural activation across a range of populations (e.g., young vs. old, depressed vs. non-depressed, etc.). This general experimental approach has great potential to further our understanding of the cognitive and neural mechanisms underlying individual differences in daily function.

**FIGURE 1:**
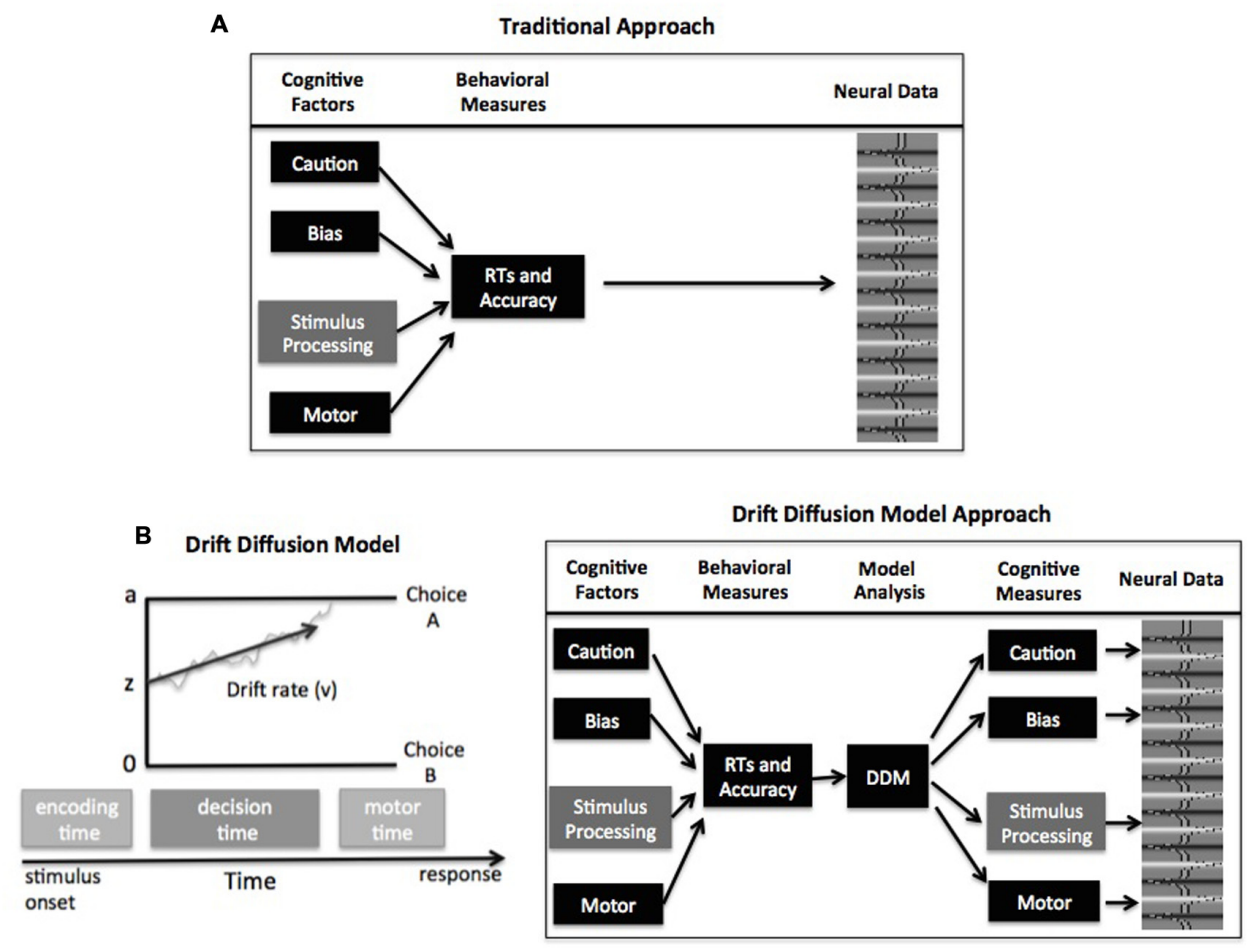
**(A)** Schematic of traditional analyses relating task performance to neural activity. **(B)** Schematic of the drift–diffusion model (DDM; left) and model-based analysis for relating cognitive mechanisms to neural activity (right). See text for description of model parameters

However, there is a potential problem to this approach that centers on the use of forward and reverse inference for relating cognitive processes to observed behavior. With forward inference, we can infer that if there is a difference in the process of interest for an experimental task, say memory processing, it should manifest as a difference in our task measures (e.g., RTs or accuracy for remembered items). To the extent that the experimental paradigms are appropriately designed, this forward inference is valid. The traditional analytical approach in studies of cognition, on the other hand, reverses this inference to claim that if there is a difference in a dependent measure like RTs, we can infer there is a difference in the process of interest like memory. This reverse inference is only valid to the extent that differences in behavior are driven by memory and *only* memory. Unfortunately, dependent measures of behavior, typically assessed by RTs and/or accuracy values, are affected by numerous processes (**Figure 1A**, top). There is robust evidence that additional factors like how cautiously a participant responds (i.e., their speed/accuracy settings) affect both RTs and accuracy (e.g., Wickelgren, 1977; Ratcliff et al., 2004). Thus if we observe individual differences in accuracy across participants in a memory task, our measure of memory processing could be contaminated by factors like response caution that are extraneous to the process of primary interest, memory. This leaves the researchers with a problem of reverse inference: differences in memory will be reflected by differences in accuracy, but that does not guarantee that differences in accuracy indicate differences in memory (Krajbich et al., 2015). This problem extends to cognitive neuroscience studies; if a researcher finds a correlation across individuals between accuracy and BOLD fMRI activity in a region of the brain, they cannot know for sure which factors are driving that relationship.

## A Model-Based Solution

Computational models of decision making present a solution to this problem. In particular, choice RT models like the drift–diffusion model (DDM; Ratcliff and McKoon, 2008) or the linear ballistic accumulator (LBA) model (Brown and Heathcote, 2008) can be used to estimate and control for individual differences in different decision components. The remainder of this commentary focuses on the DDM and LBA, but it should be noted that other accumulator models have been successful at linking behavioral and neural data (e.g., Purcell et al., 2012; Logan et al., 2015). In general, these accumulator models assume that evidence is accumulated over time until a threshold amount is reached, signaling commitment to that response option. The framework of these models contains four primary parameters that relate to different psychological components of simple decisions (**Figure 1B**): response caution is typically reflected by the boundary separation parameter (*a*, but see Cassey et al., 2014 and Rae et al., 2014 for alternative explanation) and indicates the overall amount of evidence that needs to be accumulated before the choice is committed; the duration of encoding and motor processes is reflected by the non-decision time parameter; the quality and strength of evidence from the stimulus is reflected by the drift rate parameter (*v*); and the response bias for one option over another is reflected by the starting point parameter (*z*).

In the framework of these choice RT models, the different components can all influence the dependent behavioral measures of RTs and accuracy. Fortunately, the models are mathematically specified to make predictions about expected behavior based on the values of each component, allowing these components to be estimated from the behavioral data. The general procedure for a model-based cognitive neuroscience study of individual differences is as follows: a DDM or LBA is fitted to each participant’s behavioral data to estimate values of the decision parameters (**Figure 1B**). Then these decision components can be correlated with neural data from fMRI (see Mulder et al., 2014; de Hollander et al., 2015; Forstmann and Wagenmakers, 2015 for a review), electroencephalography (EEG) (Philiastides et al., 2014; van Vugt et al., 2014; Frank et al., 2015), electromyography (EMG) (Servant et al., 2015), or single-cell recordings (Cassey et al., 2014; Hanks et al., 2014). This approach has been ubiquitously employed to describe how these decision components differ across tasks, conditions, and individuals.

## Advantages of Model-Based Analysis of Individual Differences

The analytical approach of fitting choice RT models to behavioral data offers a potential solution to the reverse inference problem when relating RTs and accuracy values to brain activity across individuals. Specifically, models like the DDM or LBA can be fitted to each participant’s data to estimate their level of response caution, quality of stimulus evidence, response bias, and non-decision processing duration. In doing so, these decision components are disentangled from each other, allowing more focused comparisons of individual differences. Thus if researchers were interested in relating individual differences in memory strength to neural activity, the drift rate parameter from the models provides a cleaner measure than RTs or accuracy values because is not influenced by extraneous factors like caution or response bias that might vary across individuals. We argue that studies of individual differences in brain activity can greatly benefit from using the decision models to control for potential confounds present in the behavioral data.

This approach provides two major advantages over traditional analyses with RTs or accuracy. First, the measure of stimulus processing which is typically of primary interest, drift rates, is not contaminated by individual differences in the other decision components. For example, in a study of lexical processing White et al., 2010b had participants perform a lexical decision task alternating between speed and accuracy emphases in the task. They found that the word frequency effect, the difference in performance between common (high frequency) and uncommon (low frequency) words, was significantly larger under accuracy emphasis compared to speed emphasis for RTs. Thus when participants were more cautious, it manifested as a larger effect in the RT measure of lexical processing, even though presumably it was driven by response caution. However, when a DDM was fitted to the data the effect of caution was absorbed by the boundary separation parameter, and the resulting drift rate estimate of lexical processing did not differ between speed and accuracy trials. In this sense the drift rate measure from the model provided a more precise index of lexical processing because it was not contaminated by differences in response caution.

Two studies with model-based analysis of neural data illustrate the advantage of this approach. We used a DDM analysis to identify the neural correlates of perceptual decision criteria in a study with fMRI (White et al., 2012). Participants classified perceptual stimuli as small or large based on different midpoints or criteria, and the analysis centered on identifying where these criteria were represented in the brain. The DDM was employed to estimate the values of the criteria, which were reflected by the drift rates, for each participant and condition to capture the variability across individuals. Although a traditional analysis that did not account for individual differences in criteria placement resulted in no significant BOLD activation, the DDM-based analysis revealed activation of the left inferior temporal gyrus relating to the changing perceptual criteria. In a similar manner, Mulder et al. (2012) used different cue conditions to bias perceptual decisions in an fMRI study. A traditional analysis of the cue conditions did not reveal any significant BOLD activity related to the bias manipulation, but an LBA-based analysis of the BOLD data that accounted for individual differences in bias revealed activation in the orbitofrontal cortex related to the perceptual bias. These two studies illustrate how the model parameters from the DDM and LBA can provide a more precise measure of individual differences in decision components and enhance the ability to relate these constructs to neural activity. Without the model-based approach to the fMRI data, both studies would have resulted in null effects in the BOLD signal.

**FIGURE 2:**
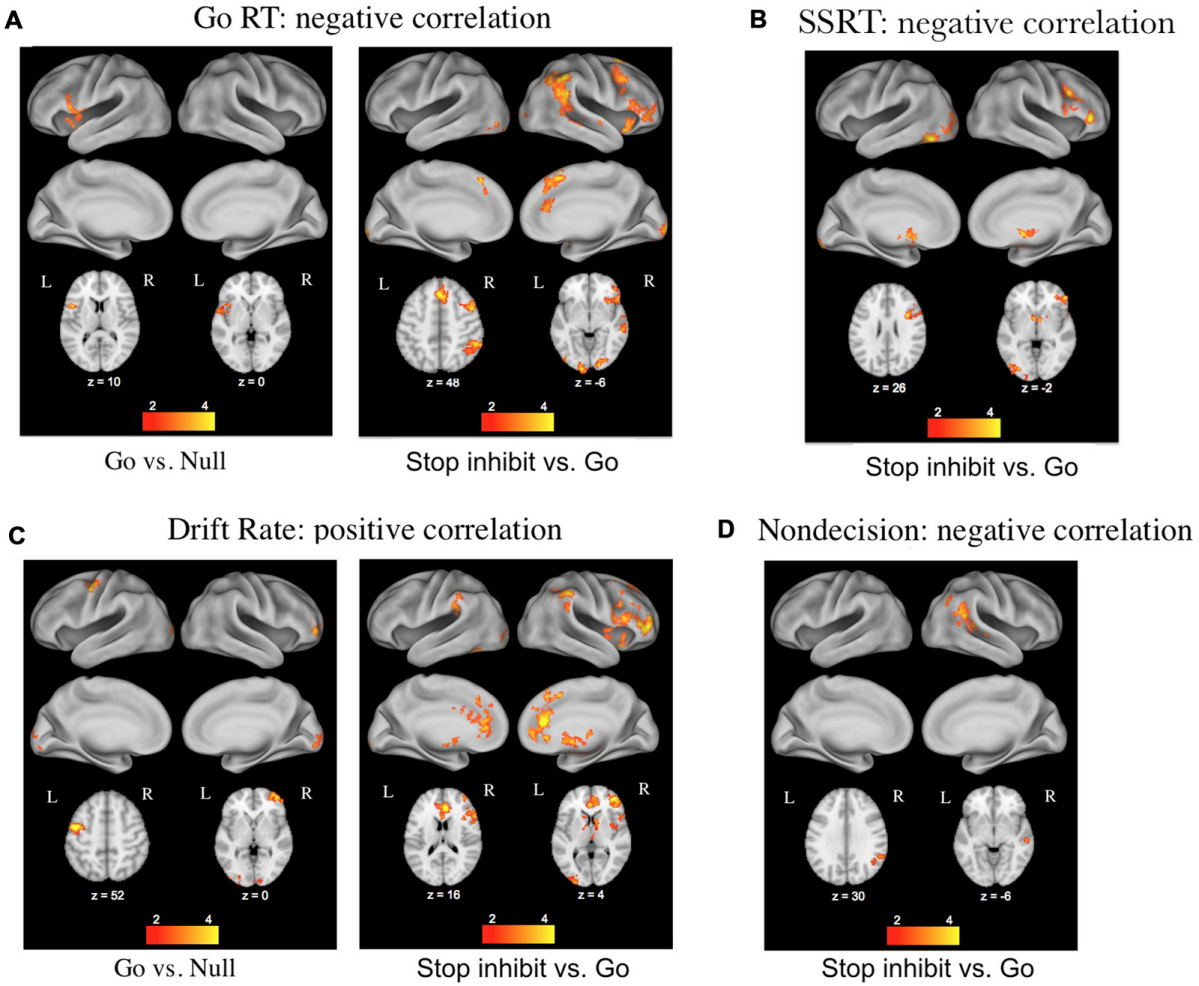
**Results of a DDM analysis of individual differences in functional magnetic resonance imaging (fMRI) activation related to performance on the stop-signal task (figure taken from White et al., 2014). (A)** Correlations with mean reaction times (RTs) from go trials. **(B)** Correlations with stop-signal reaction times from stop trials. **(C)** Correlations with the DDM parameter of drift rate on go and stop trials. **(D)** Correlations with the DDM parameter of non-decision time on stop trials.

The second major advantage of using these decision models to relate cognitive processes to brain activity stems from the ability to separately investigate the effects of differences in caution, non-decision time, bias, and drift rates across individuals. White et al. (2014) used this approach with data from a stop-signal task while participants underwent fMRI, and found that individual differences in the drift rate parameter correlated with BOLD activity in regions associated with the stopping network (pre-supplemental motor area, inferior frontal gyrus, and basal ganglia), whereas differences in the non-decision time parameter correlated with BOLD activity in the right angular gyrus of the posterior lobe (**Figure 2**). Thus the model-based approach allowed the effects of the different parameters to be dissociated, providing a more detailed examination of corresponding brain activity.

Overall, using choice RT models to relate individual differences in behavior, cognitive processes, and neural activity can enhance our study of the brain–behavior relationship. Compared to behavioral measures of RT and accuracy, the model parameters are more sensitive to the processes of interest because effects of other components are controlled by the other parameters (see White et al., 2010b, 2014). The model parameters also provide better specificity because the effects of different decision components are disentangled and can be separately investigated. This general approach has been successfully applied across a range of studies with fMRI to investigate the neural correlates of reward- and-expectation-based response bias (Mulder et al., 2012), evidence accumulation across different response modalities (Ho et al., 2009), perceptual decision criteria (White et al., 2012), and adjustments in response caution (Mansfield et al., 2011; van Maanen et al., 2011; Ho et al., 2012). We argue that such model-based analysis of neural data has particular promise for investigations of individual/group differences because populations might differ in multiple decision components (e.g., drift rates and boundary separation), and these differences must be accounted for when probing neural activity.

The use of choice RT models to relate decision components to neural activity has the additional advantage that it can be added to future and existing studies with relatively little additional work. RT data that have been previously collected can be analyzed with these models in a *post hoc* fashion, and future studies require few, if any, adjustments to the experimental design to result in data that are suitable for this type of analysis. Further, statistical packages have been designed in R (Wagenmakers et al., 2007), MATLAB (Vandekerckhove and Tuerlinckx, 2008), and Python (Wiecki et al., 2013) to implement these analyses in a user-friendly manner. Thus cognitive neuroscience studies can be enhanced by simply adding the model-based analysis to the pre-established analysis pipeline. However, there are several considerations and concerns that must be addressed for this type of model-based cognitive neuroscience, which are described below.

## Considerations for Model-Based Cognitive Neuroscience

There are myriad concerns for employing models like the DDM or LBA to relate behavior to underlying cognitive processes. Given the restricted scope of this commentary, we will focus only on the most pressing consideration here and point interested readers to excellent overviews by de Hollander et al. (2015) and Forstmann and Wagenmakers (2015) for additional information. The primary concern for performing model-based studies of individual differences in neural activity is to ensure that the parameters estimated from the model provide accurate measures of the different decision components for each participant. This involves two related concerns: first that the model assumptions are appropriate for the experimental task, and second that there are a sufficient number of observations to constrain the estimated parameters. The latter concern is especially relevant for studies of individual differences in brain activity because there are often limits to how many observations can be collected due to practical constraints like the cost of scanning time for fMRI, and the focus on individual differences disallows the practice of pooling data across participants.

Assessing the appropriateness of the model for behavioral data is typically conducted by ensuring that the model “fits” the data. This can be done by simulating data from the best-fitting parameters and comparing them to the observed data to check for concordance. If the predicted data from the model align with the observed data, it provides more confidence that the model is appropriate for the task. The DDM and LBA have been shown to successfully account for data from a range of tasks, including recognition memory (Criss, 2010; Starns and Ratcliff, 2014), lexical decision (Ratcliff et al., 2004; Wagenmakers et al., 2008), perceptual processes (Ratcliff and Rouder, 1998), inhibitory control (Cohen-Gilbert et al., 2014; White et al., 2014), and emotional classification (White et al., 2015).

However, other tasks exist that do not match the assumptions of the standard models. Specifically, tasks in which the decision evidence changes over the course of a trial, such as executive function and conflict tasks, are inconsistent with the standard assumption of a constant drift rate in the DDM and LBA. For tasks like these, augmented versions of the DDM and LBA can be created to capture the nature of the time-varying decision evidence. For example, DDMs for conflict tasks have been developed to capture the effects of engaging executive function (Hubner et al., 2010; White et al., 2011; Ulrich et al., 2015), and LBA models have been developed to account for changing decision evidence (Holmes et al., 2016). Overall, it is crucial to establish that the model assumptions are appropriate for the data being analyzed, otherwise the estimated parameter values lack validity and should not be correlated with neural activity.

A related concern about the model parameters has to do with having sufficient data to constrain the estimated parameter values. Models like the DDM and LBA require a fairly large number of observations to constrain the fitting process and result in accurate parameter recovery. This is because the parameter estimates are based on the RT distributions for correct and error trials. Thus if there are only three errors in a condition, the data are insufficient to estimate the RT distribution and constrain the parameter estimates. This is a practical concern for studies using fMRI or EEG, which are often limited in terms of the amount of data that can be collected. Fortunately there are techniques for dealing with limited data, including using filler conditions with many observations to constrain parameter estimates for conditions with few observations (White et al, 2010a), employing a Hierarchical Bayesian version of the models for situations with sparse or missing data (Turner et al., 2013a,b; Wiecki et al., 2013) and collecting additional behavioral data outside of the MRI or EEG session to increase the number of observations (Mulder et al., 2012; White et al., 2012).

Overall, model-based studies of neural activity must ensure that the parameter values estimated from the data are valid indices of the components of interest. It is incumbent upon researchers to demonstrate that the estimated parameters are valid indices of the underlying decision components by ensuring that (i) the model assumptions are appropriate for the task data to which it is applied (e.g., the model fits the data), and (ii) there are sufficient observations to constrain the model fitting process. Fortunately, there are a range of tasks and analytical approaches to ensure the success of these methods.

## Conclusion

Choice RT models like the DDM and LBA provide an elegant analytical approach to relating individual differences in cognitive processes to neural activity. Compared to traditional analyses with RTs or accuracy, the model-based analyses can provide greater sensitivity for observing individual differences in neural activation, and greater specificity for relating these differences to specific cognitive components of task performance. These advantages are particularly relevant for comparing neural activity across individuals or groups that might differ in more than one component (e.g., response caution and memory strength). Such analyses can be readily added to most existing and future studies, and have great potential to enhance the process of relating individual or group differences in behavior, neural processes, and cognitive mechanisms.

## Author Contributions

CW was responsible for drafting the manuscript and organizing the content. RC and JS were both responsible for creating the figures, editing the manuscript, developing and organizing the references. All the three authors were responsible for developing the theoretical aspects of the perspective.

## Conflict of Interest Statement

The authors declare that the research was conducted in the absence of any commercial or financial relationships that could be construed as a potential conflict of interest.
